# Dynamic Views of the Fc Region of Immunoglobulin G Provided by Experimental and Computational Observations

**DOI:** 10.3390/antib8030039

**Published:** 2019-07-01

**Authors:** Saeko Yanaka, Rina Yogo, Rintaro Inoue, Masaaki Sugiyama, Satoru G. Itoh, Hisashi Okumura, Yohei Miyanoiri, Hirokazu Yagi, Tadashi Satoh, Takumi Yamaguchi, Koichi Kato

**Affiliations:** 1 Exploratory Research Center on Life and Living Systems (ExCELLS) and Institute for Molecular Science (IMS), National Institutes of Natural Sciences, 5-1 Higashiyama, Myodaiji, Okazaki 444-8787, Japan; 2 Graduate School of Pharmaceutical Sciences, Nagoya City University, 3-1 Tanabe-dori, Mizuho-ku, Nagoya, Aichi 467-8603, Japan; 3 Department of Functional Molecular Science, SOKENDAI (The Graduate University for Advanced Studies), Okazaki, Aichi 444-8787, Japan; 4 Department of Structural Molecular Science, SOKENDAI (The Graduate University for Advanced Studies), Okazaki, Aichi 444-8585, Japan; 5 Institute for Integrated Radiation and Nuclear Science, Kyoto University, 2-1010 Asashiro-Nishi, Kumatori, Osaka 590-0494, Japan; 6 Institute for Protein Research, Osaka University, 3-2 Yamadaoka, Suita, Osaka 565-0871, Japan; 7 School of Materials Science, Japan Advanced Institute of Science and Technology (JAIST), 1-1 Asahidai, Nomi 923-1292, Japan

**Keywords:** Immunoglobulin G, Fc, conformational dynamics, molecular dynamics simulation, small-angle X-ray scattering, nuclear magnetic resonance, N-glycan, core fucosylation

## Abstract

The Fc portion of immunoglobulin G (IgG) is a horseshoe-shaped homodimer, which interacts with various effector proteins, including Fcγ receptors (FcγRs). These interactions are critically dependent on the pair of N-glycans packed between the two C_H_2 domains. Fucosylation of these N-glycans negatively affects human IgG1-FcγRIIIa interaction. The IgG1-Fc crystal structures mostly exhibit asymmetric quaternary conformations with divergent orientations of C_H_2 with respect to C_H_3. We aimed to provide dynamic views of IgG1-Fc by performing long-timescale molecular dynamics (MD) simulations, which were experimentally validated by small-angle X-ray scattering and nuclear magnetic resonance spectroscopy. Our simulation results indicated that the dynamic conformational ensembles of Fc encompass most of the previously reported crystal structures determined in both free and complex forms, although the major Fc conformers in solution exhibited almost symmetric, stouter quaternary structures, unlike the crystal structures. Furthermore, the MD simulations suggested that the N-glycans restrict the motional freedom of C_H_2 and endow quaternary-structure plasticity through multiple intramolecular interaction networks. Moreover, the fucosylation of these N-glycans restricts the conformational freedom of the proximal tyrosine residue of functional importance, thereby precluding its interaction with FcγRIIIa. The dynamic views of Fc will provide opportunities to control the IgG interactions for developing therapeutic antibodies.

## 1. Introduction

Antibodies play pivotal roles in the immune system as multifunctional glycoproteins, coupling between antigen recognition and effector functions, as typified by immunoglobulin G (IgG). The Fab region of each IgG binds to its specific antigen while the Fc region interacts with effector proteins, including the complement component C1q and a series of Fcγ receptors (FcγRs) [1,2,3,4]. Further, the Fc portion of IgG interacts with various proteins other than the effector proteins, such as staphylococcal protein A and streptococcal protein G [5]. This versatile functionality of IgG and the other classes of antibodies is believed to be attributed to their structural flexibility and plasticity [6,7].

Antibodies have modular structures, in which the Ig-fold domains as building blocks are connected through flexible linkers. In IgG, the two identical light chains are each divided into the V_L_ and C_L_ domains, whilst the two identical heavy chains are each composed of the V_H_, C_H_1, C_H_2, and C_H_3 domains. The C_H_1 and C_H_2 domains are separated by the hinge region, which possesses significant degrees of freedom for internal flexibility.

Each Ig domain is structurally characterized by nine or seven β strands (in the V and C domains, respectively) connected by turn or loop segments. The hypervariable loops in the V_L_ and V_H_ domains are directly involved in antigen binding. In particular, the third hypervariable loop in the V_H_ domain (H3 loop) is extremely divergent in amino acid sequence and it exhibits dynamic conformational multiplicity [8,9,10]. Antigen binding generally renders the hypervariable loops less mobile. The conformational plasticity of the hypervariable loops is the key property for the antigen recognition mechanism. Kinetic data have shown that the antibody combining site undergoes conformational changes upon interacting with the antigen, thereby involving conformational selection as well as induced-fit processes [11]. Antigen binding often induces non-local conformational rearrangements of the V_L_ and V_H_ domains, thereby altering their orientation relative to each other and with respect to the C_L_ and C_H_1 domains [4,12]. It has been speculated that such allosteric effects are involved in functional cooperativity between antigen binding and interaction with the effector proteins [4].

Dynamic views of antibody structures thus offer deep insights into their functional mechanisms. Dynamic structures of antibodies in solution have recently been characterized by sophisticated experimental techniques, including nuclear magnetic resonance (NMR) spectroscopy [13,14], solution scattering [15,16,17,18,19], cryo-electron microscopy [7,20], and high-speed atomic force microscopy [21]. In addition, computational approaches offer powerful tools for providing dynamic views of antibody structures [22]. These techniques highlight considerable variability in the spatial arrangements of the two Fab arms with respect to the Fc stem in IgG, which is provided by the conformational freedom of the hinge region, facilitating IgG’s bivalent binding to antigens with various spatial arrangements, such as bacterial surfaces.

By contrast to the extensively characterized Fab and hinge dynamics, the dynamic properties of the IgG-Fc structure in solution remain to be fully understood, although the crystal structures of Fc exhibit significant conformational variability [23,24,25]. In addition, the IgG-Fc possesses a conserved glycosylation site at Asn297 in each C_H_2 domain, at which a bi-antennary complex-type oligosaccharide is present with heterogeneity in sequence and conformation [26,27]. This N-glycosylation is critically important for IgG interactions with the effector proteins and the consequent effector functions [3,28,29]. Deglycosylation of IgG-Fc impairs its binding to C1q and FcγRs. In contrast, the removal of the core fucose residue from the N-glycan of human IgG1-Fc results in enhancement of its interaction with FcγRIIIa, thereby dramatically improving the antibody-dependent cell-mediated cytotoxicity (ADCC) [30,31,32]. Therefore, in this study, we have attempted to provide dynamic views of the Fc region of human IgG1 by computational approaches with experimental validation to discuss their functional significance. In particular, we will shed light on intramolecular interaction networks involving the N-glycans, providing mechanistic insights into the improved efficacy of the therapeutic antibodies by the glycan remodeling.

## 2. Materials and Methods

### 2.1. Molecular Dynamics Simulation

The starting structures were built on the basis of the crystal structure Protein Data Bank (PDB) entry 3AVE, 5IW3, and 2DTS, supplemented with crystallographically unobserved segments in each structure as follows: In 3AVE and 2DTS, N-terminal segments (T224–E233 in chain A and T224–G236 in chain B), C-terminal segments (P445–K447), and the terminal galactose residues of the α1-6Man branches; and in 5IW3, N-terminal segments (T224–L235), C-terminal segments (S444–K447), and the core fucose residue. The crystallographically unobserved galactose residues of 3AVE and 2DTS were modeled by superposition of their GlcNAc residues of the α1-6Man branches and the corresponding Gal-β1,4-GlcNAc disaccharide moieties of an sFcγRIIIa-bound Fc crystal structure (3AY4). The core fucose residues of 5IW3 were modeled by superposition of its reducing terminal GlcNAc residues and the corresponding Fuc-α1,6-GlcNAc disaccharide moieties of 3AVE. In the 5IW3-based model, E379 and M381 were substituted in silico with aspartate and leucine, respectively, by using PyMOL (https://www.pymol.org). The AMBER14 [33] program package was used with the force fields AMBER ff14SB [34] and GLYCAM06 [35] for proteins and glycans, respectively, along with the TIP3P water model [36]. The IgG1-Fc models derived from 3AVE, 5IW3, and 2DTS were placed in boxes containing 28,135, 24,372, and 28,243 water molecules, respectively. After the preparation of the initial conformations, we performed equilibrium simulations for 4 ns with NPT ensemble with periodic boundary conditions. The system was heated from 5 to 310 K for 100 ps at a constant pressure of 1 atm. Eight simulations were then performed with different velocities. Each simulation time period was 400 ns. All production simulations were done at 300 K with the weak-coupling algorithm in the NPT ensemble. The bonds involving hydrogen atoms were constrained by the SHAKE algorithm [37]. The electrostatic interactions were treated with the particle mesh Ewald method and the cutoff distance for the non-bonded interactions was 8.0 Å. The first 80 ns were removed considering the time needed for the initial structure to reach equilibrium. Molecular dynamics (MD) simulations for the Fc fragments without the hinge and without the N-glycans were performed using the same protocol and initial models based on 3AVE (with 19,410 water molecules) and 5IW3 (with 24,551 water molecules), respectively. Ensemble models were created from 25,600 conformers extracted every 100 ps from the 320 ns simulation results of the eight production runs. The root mean square fluctuation (RMSF) for each amino acid Cα atom of IgG1-Fc was calculated from 3,200 conformers extracted from each of the eight production runs, which were superimposed by the Cα atoms, yielding an average structure. The RMSF was derived on the average structure. 

### 2.2. Sample Preparation

For the small-angle X-ray scattering (SAXS) measurements, the Fc fragment with core fucosylation was cleaved from commercially available IgG1 antibodies (Chugai Pharmaceutical, Tokyo, Japan). For the NMR measurements, the fucosyl Fc fragment was cleaved from IgG1 metabolically labeled with [CO, α, β, γ, ε1, ε2-^13^C_6_; β2, δ1, δ2-^2^H_3_; ^15^N] tyrosine (Taiyo Nippon Sanso, Tokyo, Japan). The metabolic isotope labeling, proteolytic digestion, and purification of the Fc fragments were performed according to previously reported protocols [13,38]. 

### 2.3. Small-Angle X-Ray Scattering

SAXS experiments were performed using Fc dissolved in 50 mM Tris-HCl, pH.8.0, 150 mM NaCl at a protein concentration of 5.0 mg/mL with NANOPIX (Rigaku, Tokyo, Japan) at 25 °C. X-rays from a high-brilliance point-focused X-ray generator (MicroMAX-007HF, Rigaku, Tokyo, Japan) were focused with a confocal mirror (OptiSAXS) and collimated with a confocal multilayer mirror and two pinholes collimation system with the lower parasitic scattering, “ClearPinhole”, supplied for the X-rays with the flux of 2.0 × 10^8^ cps at the sample position (high flux mode). The scattered X-rays were detected using a two-dimensional semiconductor detector (HyPix-6000, Rigaku, Tokyo, Japan) having the spatial resolution of 100 μm. By measuring SAXS profiles in two sample-to-detector distance (SDD) conditions, 1320 mm and 300 mm, the wide *Q*-range (0.015 Å^−1^–0.5 Å^−1^) were covered. In addition, for removal of unfavorable aggregates from the sample solution, the laboratory-based SEC-SAXS System (LA-SSS) was employed to measure the SAXS profile in the lower-Q range (0.015 Å^−1^–0.08 Å^−1^, SDD = 1320 mm). 

The theoretical SAXS profiles were independently calculated from the atomic coordinates of the ensemble model containing 25,600 conformers. Finally, an MD-derived SAXS profile was obtained by averaging the 25,600 calculating SAXS profiles. The χ^2^ values for the-goodness-of-fit is defined as follows:

$$\chi^{2}=\frac{1}{N-1}\underset{j}{\sum }{\left[\frac{I_{\exp}\left(q_{j}\right)-cI_{\mathrm{calc}}\left(q_{j}\right)}{\sigma \left(q_{j}\right)}\right]}^{2}$$

where *N*, *I*_exp_(*q*), σ(*q*), *I*_calc_(*q*), *c* is the number of experimental data points, the experimental scattering intensity, its error, the calculated scattering intensity and the scaling factor, respectively.

### 2.4. NMR Measurement

Two-dimensional heteronuclear single-quantum correlation nuclear Overhauser effect spectroscopy (HSQC-NOESY) spectra were acquired for fucosylated IgG1-Fc labeled with [CO, α, β, γ, ε1, ε2-^13^C_6_; β2, δ1, δ2-^2^H_3_; ^15^N] tyrosine and dissolved in 5 mM sodium phosphate buffer, pH 6.0, containing 50 mM NaCl and 10% D_2_O at a protein concentration of 10 mg/mL by using an AVANCEIII 950 spectrometer (Bruker BioSpin) equipped with a TCI cryogenic probe at 300 K. The NMR spectral data were recorded at a proton observation frequency of 950.3 MHz with 128(*t*_1_) × 2048(*t*_2_) complex points and 512 scans per *t*_1_ increment, with a mixing time of 300 ms.

## 3. Results and Discussion

### 3.1. Overall Conformation of IgG-Fc

**Figure 1:**
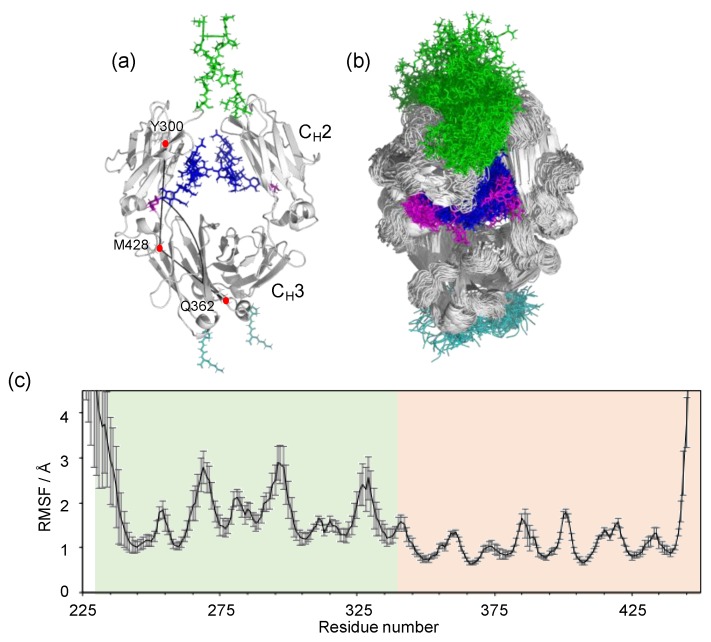
MD simulation of IgG1-Fc. (**a**) The starting structure of the MD simulation, based on the crystal structure of fucosyl IgG1-Fc (3AVE) supplemented with the hinge (green; T224–E233 in chain A and T224–G236 in chain B) and C-terminal (cyan; P445–K447) segments along with the terminal galactose residues (magenta) of the α1-6Man branches. The N-glycans are colored blue except for the terminal galactose. The intra-chain domain-orientation angle between C_H_2 and C_H_3 defined by Cα atoms of Y300, M428, and Q362 are shown in chain A. (**b**) The superposition of 256 structures extracted every 100 ns from the MD trajectory. The structures were visualized by PyMOL (https://www.pymol.org). (**c**) The RMSF for each amino-acid Cα atom of IgG1-Fc, which was calculated as described in Materials and Methods. White, hinge; light green, C_H_2; light orange, C_H_3.

The Fc portion of IgG has a horseshoe-shape, harboring a pair of N-glycans packed between the two C_H_2 domains, which consequently make no direct contacts, while the C_H_3 domains extensively contact each other (Figure 1a). This domain arrangement renders the C_H_2 domains more mobile than the C_H_3 domains as implicated by the crystallographic data [23,24,25]. The *B* factors of the C_H_2 domains are generally higher than those of the C_H_3 domains [39]. Moreover, the C_H_2 domains exhibit divergent orientations in crystal structures in free and liganded states and in various glycoforms. Indeed, the great majority of the IgG1-Fc crystal structures deposited in Protein Data Bank (PDB) exhibit asymmetric quaternary structures even in uncomplexed states, with few exceptions, for example, 5IW3 with a crystallographic two-fold axis. However, these conformational deformations might be, at least partially, ascribed to non-physiological crystal contacts. Frank et al. have performed a 200 ns MD simulation of human IgG1-Fc with fully galactosylated glycans and demonstrated that the C_H_2 domains showed significant degrees of motional freedom [24]. In general, MD simulation results depend on the calculation protocol, including the initial structure and simulation time as well as the force field.

We performed long-timescale MD simulations in explicit water, using our determined crystal structure of human IgG1-Fc (3AVE) [40] as the initial model. We attempt to deal with a major glycoform of Fc, in which two complex-type N-glycans are mono-galactosylated at the α1-6Man branch. The crystal structure was supplemented with models of the hinge and the C-terminal regions along with the non-reducing terminal galactose residues in the α1-6Man branches, because these parts gave no interpretable electron density in this crystal structure (Figure 1a). From the MD trajectories (2.56 μs in total for each Fc glycoform), 25,600 conformers were extracted to create an ensemble model reproducing possible conformational spaces of the Fc glycoproteins.

**Figure 2:**
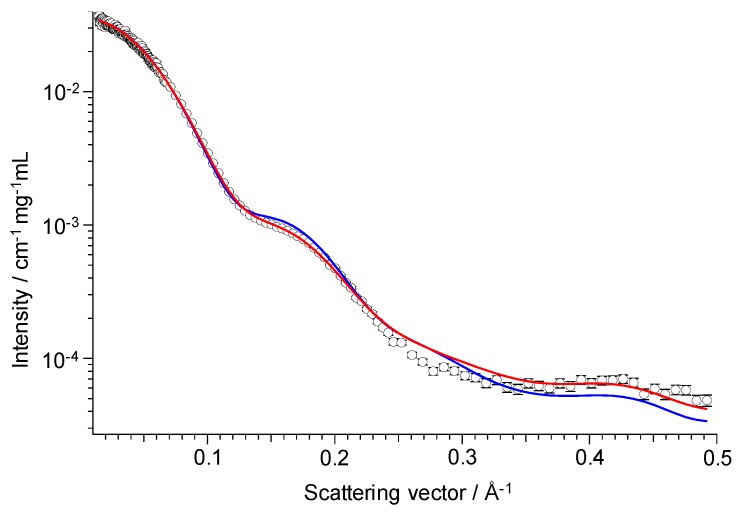
SAXS-based experimental validation of the MD-derived ensemble model. SAXS profile of fucosyl IgG1-Fc (open circle) shown with theoretical profiles computed from the MD-derived ensemble model (red) and the crystal structure (3AVE) (blue).

For experimental validation of the simulation results, we measured SAXS of the Fc region, which has been applied for the characterization of Fc structures in solution [41,42,43]. The SAXS profile computed from the Fc ensemble model was in good agreement with the experimentally obtained SAXS profile (Figure 2). The MD-derived SAXS profile reproduced the experimental data (χ^2^ = 6.8) better than did a profile computed from the crystal structure used for building the initial model (χ^2^ = 13.6). The smallest χ^2^ value, 6.8, was achieved with the ensemble model created from the total 2.56 μs MD simulation, while the χ^2^ value calculated for each of the eight production runs were larger (range 7.3–32.2), suggesting that a shorter MD simulation was not enough for exploring the Fc conformational space.

**Figure 3:**
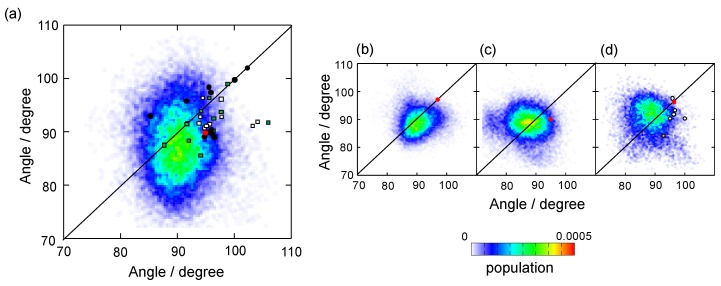
Distribution of intra-chain domain-orientation angles between C_H_2 and C_H_3 for the ensemble models of IgG1-Fc and various crystal structures of IgG1-Fc. The angles between the C_H_2 and C_H_3 domains of chain A and chain B were plotted on the X axis and Y axis, respectively, for the ensemble models derived from MD simulations starting from the initial structures based on (**a**) asymmetric crystal structure (3AVE) supplemented with the crystallographically unobserved N- and C-terminal segments, (**b**) symmetric crystal structure (5IW3) supplemented with crystallographically unobserved N- and C-terminal segments, (**c**) crystallographically observed parts of 3AVE, and (**d**) 5WI3 supplemented with the crystallographically unobserved N- and C-terminal segments with deletion of the N-glycans. In A, B, and C, the N-glycans of each initial structure were modeled to have the core fucose residue and the terminal galactose residue of the α1-6Man branch. The angles observed in the crystal structures are represented as circles for uncomplexed Fc structures (red, the starting structures used for the corresponding MD simulations; black, Fc with native N-glycans; white, Fc with enzymatically trimmed N-glycans; yellow, aglycosylated Fc), rectangles for complexed Fc structures (white, complex with sFcγRs; green, complex with other ligands).

Although our simulation results confirmed that, besides the hinge and the C-terminal segments, the C_H_2 domains exhibit considerable motion in comparison with the C_H_3 domains (Figure 1b,c), the major conformers exhibited almost symmetric structures with C_H_2-C_H_3 angles (approximately 90 degrees) that were more acute in comparison with that of the uncomplexed Fc crystal structures, including those used as the initial structures in the simulations (Figure 3). We performed an additional MD simulation by using a two-fold symmetrical Fc model (based on the crystal structure 5IW3) as the initial model. Despite the remarkably different starting model, the MD result showed a similar tendency: The MD-derived conformers still exhibited symmetrical but significantly stouter quaternary structures (Figure 3). These results were quantitatively consistent with the previously reported MD simulation of the fully galactosylated Fc [24], suggesting that the Fc packed in crystal lattices is apt to adopt asymmetric slim quaternary structures as compared with those in solution.

The experimentally validated conformational ensembles of IgG1-Fc encompassed most of the previously reported crystal structures because of the variability of the C_H_2 domains with respect to the C_H_3 domains (Figure 3). The conformational ensemble included the crystal structures of human IgG1-Fc in complex with Fc-binding proteins such as protein A domains, though they are minor conformational species. The Cα RMSD was 1.1 Å between the protein A B domain-bound Fc crystal structure (IL6X) [44] and its most resembling conformer found in the MD-derived ensemble. These findings imply that interactions of Fc with these proteins involve conformational selection mechanisms. However, the asymmetrically deformed Fc structures bound to sFcγRIIa were obviously far from the MD-derived conformational cluster and very rarely found in the corresponding ensemble model: The Cα RMSD was 3.2 Å between the sFcγRIIa-bound Fc crystal structure (3RY6) [45] and its most resembling conformer found in the MD-derived ensemble (excluding the Asn286–Gln295 segments because of inconsistent interpretation of their electron densities). This finding suggested that induced-fit mechanisms are involved in the binding process.

The two C_H_2 domains are tethered at their N-termini through the disulfide-linked hinge region and they bracket the pair of N-linked oligosaccharides. Therefore, it was highly plausible that the hinge and the glycans critically affect the conformational space of the Fc region. Indeed, the Fc crystal structures of IgG1-Fc with different glycoforms showed different quaternary conformations [23]. We performed MD simulations of IgG1-Fc without the hinge region and that of IgG1-Fc without the N-glycans. Elimination of these parts resulted in greater degrees of motional freedom of the C_H_2 domains with increases in the population of extremely asymmetric quaternary conformations. This is seemingly inconsistent with a crystal structure of aglycosyl Fc reported by Borrok et al., which adopted a more closed conformation [41]. However, in that study, they suggested that aglycosyl Fc assumes a more open C_H_2 orientation based on their SAXS observation, which was apparently consistent with our MD simulation results. The asymmetrically distorted quaternary conformation was found in the previously reported MD simulation of a hinge-truncated Fc [24], which is also qualitatively consistent with our data.

It has been reported that either disulfide cleavage at the hinge or deglycosylation of Fc impairs the interactions of IgG1 with the effector proteins [2,3]. In addition to the local conformational perturbations suggested by NMR studies [14,46,47], the increased mobility of the C_H_2 domains may negatively contribute to the affinities of the IgG-Fc for the effector proteins, at least partially, due to the increase in the conformational entropic penalty upon their binding. 

### 3.2. Intramolecular Interaction Networks of N-Glycans

In general, carbohydrate chains are conformationally dynamic compared to polypeptide chains and, therefore, yield ambiguous electron densities in the crystal structures of glycoproteins [48,49]. However, this is not true for the N-glycans of IgG-Fc because they are packed between the two C_H_2 domains and, therefore, are restricted in terms of internal motion, which has been confirmed by the previous and present MD simulations [24]. Consequently, in many crystal structures, the Fc N-glycans have been visualized except for the non-reducing terminal galactose residues in the α1-3Man branches, which are projected to the inner space of the horseshoe-structure and therefore are considerably mobile [14,28].

**Figure 4:**
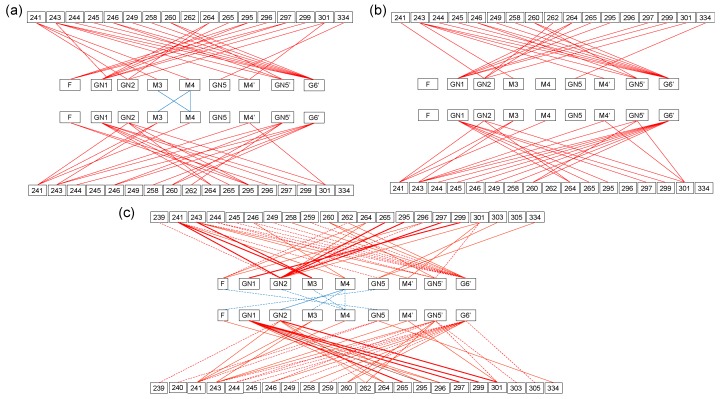
Intramolecular interaction networks of the IgG1-Fc glycans. A pair of contact residues between the N-glycan and the polypeptide chain or between the two N-glycans found within 4 Å is connected by a line segment for the crystal structures of (**a**) Fc alone (3AVE) and (**b**) sFcγRIIIa-bound Fc (5XJE). The terminal galactose residues of α1-6Man branch in 3AVE were modeled as described in the Materials and Methods. The contact pairs involving either of these galactose residues are conserved in the crystal structure 5IW3, which gave electron densities of the terminal galactose residues. (**c**) Pairs of contact residues found within 4 Å in the ensemble model derived from the MD simulation are connected by different types of line segments (red for carbohydrate-protein contact and cyan for carbohydrate-carbohydrate contact) according to incidence as follows: More than 24,000 pairs (thick solid line), 24,000 to 16,000 pairs (thin solid line), and 16,000 to 8,000 pairs (dashed line).

The crystal structures indicated that the core part and the α1-6Man branch of the N-glycans make extensive contacts with the amino acid residues located in the inner surface of the C_H_2 domain, while the interactions between the two glycans are quite limited (Figure 4) [28]. The crystallographic data of the sFcγRIIIa-bound Fc showed rearrangements of the interaction network, creating new contact pairs with concomitant loss of a number of contact pairs, resulting in the disappearance of the intramolecular glycan-glycan interactions [40,50]. All the contact pairs between the N-glycan and the C_H_2 amino acid residues in both the free and the complexed forms were found in the MD-derived ensemble model. Moreover, the ensemble model included more contact pairs not only between glycan and amino acid residues but also between the two glycans, which were not observed in the Fc crystal structures, demonstrating dynamic behaviors of the N-glycans within the inner space of the Fc. This is consistent with the previously reported NMR observation for the mobility of the terminal galactose residue of the α1-6Man branch [27] and its missing electron densities in many crystal structures exemplified by 3AVE and 2DTS [40]. All these data suggest that the pair of N-glycans not only restrict motional freedom of the C_H_2 domains but also endow quaternary structure plasticity through multiple intramolecular interaction networks. It is conceivable that the intramolecular N-glycan interaction networks critically depend on the Fc glycoform. Lee and Im reported MD simulations of human IgG1-Fc glycoforms exhibiting a series of sequentially truncated high-mannose-type glycans. In this extreme case, the N-glycans dynamically interconverted between C_H_2-bound and unbound forms and the glycan truncation affected the Fc quaternary conformational dynamics [51].

### 3.3. Effects of Fc Defucosylation of its Dynamic Conformation and FcγR Interaction

**Figure 5:**
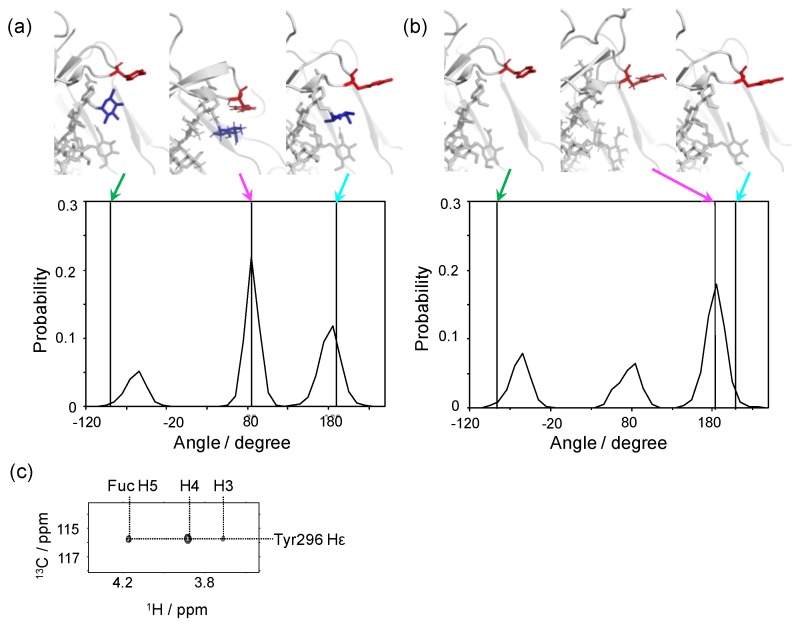
Conformational dynamics of the side chain of Tyr296 of IgG1-Fc depending on the core fucosylation. Distributions of χ1 dihedral angles of Tyr296 in the ensemble models derived from MD simulations are plotted for (**a**) fucosylated IgG1-Fc and (**b**) non-fucosylated IgG1-Fc. The typical conformational snapshots of derived from the major conformational states (magenta arrows) in the simulation trajectory are shown along with the crystal structures used for building the starting models (green arrows; A, 3AVE; B, 2DTS) and those of sFcγRIIIa-bound Fc (cyan arrows; A, 5XJE; B, 3AY4). (**c**) 2D HSQC-NOESY spectrum of IgG1-Fc labeled with [CO, α, β, γ, ε1, ε2-^13^C_6_; β2, δ1, δ2-^2^H_3_; ^15^N] tyrosine.

The major forms of IgG1-Fc N-glycans share the core fucose residue, which negatively contributes to binding to FcγRIIIa, and thereby impairs ADCC activity [30,31,32]. While our crystallographic data have indicated that the core fucosylation does not affect the overall conformation of IgG1-Fc, our NMR data have shown defucosylation-induced microenvironmental changes surrounding this fucose residue. This is best exemplified by Tyr296, which is crucially involved in the interaction with FcγRs, including FcγRIIIa [40,52]. We performed a long-timescale MD simulation starting from a model based on a crystal structure of non-fucosylated IgG1-Fc (2DTS) for comparing the local conformation of this tyrosine residue between the fucosylated and non-fucosylated glycoforms (Figure 5). In either of the crystal structures, where free IgG1-Fc in fucosylated or non-fucosylated form was used as the initial structure in the simulation, the Tyr296 side chain adopts a semi-outward conformation with χ1 dihedral angles of −96 and −91 degrees, respectively [40]. Remarkably, these conformations were not the major ones during our MD simulation. In the fucosyl form, the most populated conformer of the side chain of Tyr296 stays in a “flipped-in” state with a χ1 angle of approximately 80 degrees, making contacts with the core fucose. This inward conformation has been experimentally confirmed based on the observation of nuclear Overhauser effect (NOE) connectivities between the core fucose and the Tyr296 side chain (Figure 5c). By contrast, in the non-fucosyl form, the conformational state of this tyrosine is more divergent with a significantly increased outward conformation with a χ1 angle of approximately 180 degrees. The conformational multiplicity of Tyr296 in the non-fucosyl form was also indicated by our NMR data. Interestingly, the crystallographic data indicated that Tyr296 is involved in the interaction with FcγRIIIa in a flipped-out state with a χ1 angle of 189 and 200 degrees in the fucosylated and non-fucosylated glycoforms, respectively [50,53]. These data indicated that the Tyr296 side chain is stabilized in the inward conformation through interaction with the core fucose and, on defucosylation, undergoes a conformational population shift with an increased outward conformation, which is favorable for FcγRIIIa binding. It is possible that, in the fucosylated IgG1, the flipping-out of this tyrosine side chain can be a rate-limiting step in its interaction with FcγRIIIa. This view is consistent with the previously reported kinetic data indicating that the core fucosylation of IgG1 primarily affects its association phase of FcγRIIIa binding [54].

## 4. Conclusions

In this study, the conformational spaces in IgG1-Fc glycoproteins were investigated by performing long-timescale MD simulations in explicit water with validation by experiments in solution. The MD-derived conformational ensembles included most of the crystallographic snapshots of IgG1-Fc thus far reported, whilst its conformational space was restricted by the hinge disulfides and the Asn297 glycans. We presumed that during the evolutionary process, the freedom of the Fc quaternary conformation was optimally restricted to strike a balance between the increase in adaptability to a variety of binding partners and the decrease in a conformational entropic penalty upon their interactions. From the viewpoint of antibody engineering, the conformational plasticity of Fc can be targeted to control its interactions with specific binding partners. As in the case of the removal of the core fucose, engineering strategies have been developed to control local conformational dynamics around the specific target binding sites of the Fc region, through amino acid substitutions and/or N-glycan remodeling [30,31,32,55,56]. In a complementary approach, a detailed understanding of the quaternary conformational dynamics of the Fc region, through concerted theoretical and experimental approaches will open up opportunities for developing novel therapeutic antibodies by allosteric control of their interactions with effector molecules.

## Abbreviations

ADCC	Antibody-dependent cell-mediated cytotoxicity
FcγR	Fcγ receptor
Fuc	Fucose
Gal	Galactose
GlcNAc	*N*- Acetylglucosamine
HSQC	Heteronuclear single-quantum correlation
IgG	Immunoglobulin G
Man	Mannose
MD	Molecular dynamics
NMR	Nuclear magnetic resonance
NOE	Nuclear Overhauser effect
NOESY	Nuclear Overhauser effect spectroscopy
PDB	Protein Data Bank
RMSD	Root mean square deviation
RMSF	Root mean square fluctuation
SAXS	Small-angle X-ray scattering
